# A Treatise on the Venereal Diseases of the Eye

**Published:** 1831-01-01

**Authors:** 


### A Treatise on the Venereal Diseases of the Eye. By *William Lawrence*, F.R.S., &c. &c.

[Second and concluding Article.]

In a former article we considered the gonorrhœal diseases of the eye, and the present will be dedicated to its syphilitic affections. These comprise, 1st: syphilitic iritis; and 2ndly, syphilitic ulceration of the eye-lids.

### I. Syphilitic Iritis; Iritis Syphilitica.

The iris is liable to inflammation from various causes, of which syphilis is perhaps the most frequent. A description of the origin, progress, and consequences of the syphilitic form will nearly serve for those of all the rest.

The membrane lining the chambers of the aqueous humour resembles that of the serous cavities, and in it, as in them, inflammation is attended by effusion of albuminous fluid or coagulating lymph. The structure and situation of the texture affected will account for our not observing the four ordinary concomitants of inflammation, swelling, redness, heat, and pain. But effusion is a common occurrence, and is indiscriminately known as coagulable lymph. It changes the colour and appearance of the iris, impairs and destroys its motions, occasions adhesions to the surrounding parts, alters the form and size of the pupil, and produces more or less destruction of vision. Iritis is an adhesive inflammation. It is attended with enlargement of the vessels of the sclerotic, and consequently by preternatural redness of the eye, increased sensibility to light, and lachrymal discharge. Such are the general characters of iritis. We next proceed to consider the symptoms more in detail. And first of—

*Change of Colour in the Iris.* This is one of its most striking characters. A light-coloured iris becomes, under inflammation, yellowish or greenish, occasionally distinctly yellow; if the eye be blue, it sometimes assumes a bright green tint. Generally the tint, of whatever colour, is of a dull and muddy cast, and darker than natural. A dark iris, when inflamed, presents a reddish tinge. There is a complete loss of the natural brilliancy of the iris, it grows dull and dark, and its beautiful fibrous arrangement is either confused or entirely lost. These changes commence in the pupillary margin. In the early stage the very edge of the pupil only may be affected, the internal circle is then altered in colour and thickened, and afterwards the external or ciliary edge is implicated. The alteration of colour is produced by effusion into the texture of the organ, and is such as would arise from blending the yellowish or brownish colour of the lymph with the natural hue of the iris.

*Various Appearances of the Effused Lymph.* The deposition of lymph may be variously modified: 1st. It may be effused into the iris, producing the changes of colour alluded to. 2dly. It may form a thin layer, covering a larger or smaller surface. Thus, the edge of the pupil first, and subsequently the lesser circle, may assume a reddish brown or rusty colour in the beginning of the affection. The discoloured part has a rough villous appearance, and on looking at it closely, particularly sideways, we generally find a slight elevation and irregularity of surface produced by this new deposit. Sometimes the stratum of lymph has a light yellowish brown or ochrey tint, and a loose villous texture, rising into obviously prominent masses. The rusty colour is most common, especially in blue irides; the other is seen in the grey or orange-grey. This deposit is usually confined to the inner circle, but the outer is generally more or less discoloured and dull. 3dly. The lymph may be effused in distinct masses or tubercles, of a yellowish or reddish brown colour, sometimes of a bright red. They vary in size from that of a pin's head to that of a split-pea. Often there is only one; there may be two, or more. They may be deposited on the edge of the pupil, or in any part of the anterior surface of the iris. If the inflammation is very active or the treatment inefficient, the lymph is sometimes so abundant as nearly to fill the anterior chamber, when it has a light dirty yellowish colour, and often a loose semi-transparent texture. 4thly. Under violent inflammation coagula of blood are sometimes mixed with the tubercular masses of lymph. Mr. L. has seen this when the inflammation was not of the most violent kind. 5thly. Lymph may be poured out from the margin of the pupil or uvea, so as to agglutinate them partially or generally to the capsule of the crystalline. A mass of lymph sometimes fills the pupil; more frequently a thin greyish web stretches across the opening, which is rendered cloudy. Lymph may be effused in considerable quantity into the posterior chamber, and either make its way through the pupil into the anterior chamber, or cause a bulging of the sclerotica, penetrate that membrane, and form a tumour under the conjunctiva. The swelling in these cases has sometimes a yellowish appearance on its most prominent part, from which, with the intense redness and violent pain in the eye, suppuration of the globe has been suspected, and the part punctured. No pus has escaped. Mr. L. thinks he may assert that suppuration never takes place in syphilitic iritis, the inflammation being always adhesive, the changes produced by the effusion of lymph.

Mr. Lawrence has never seen hypopion in this disease: nor those bright yellow convex masses which burst after a time, allowing the escape of a yellow matter, which falls down in the anterior chamber. The latter have been considered as peculiar to idiopathic iritis, but our author has seen them in two or three instances in which a syphilitic origin was dubious.

The effusion into the texture of the iris, producing the general change in its appearance, and the reddish brown discoloration of its inner circle with thickening of the pupillary margin, are generally the first alterations observed; they may take place separately, but are usually conjoined. As the inflammation proceeds the tubercular masses appear. In the most violent degree, large effusion takes place into the anterior, or posterior chamber, and the pupil. As the several modifications of effusion depend on the degree of inflammation, they may be variously combined together, according to the stage of the inflammation and effects of the measures adopted. Occasionally, however, the inflammation, although violent and of long standing, is characterized by general discoloration alone, or with the addition of a thin stratum of lymph on the inner circle of the iris. In some instances the tubercular deposition of lympth takes place with hardly any other appreciable alteration.

*Motions of the Iris and State of the Pupil.* "The motions of the iris must be seriously impaired by the changes just described, more particularly by the interstitial effusion of lymph. It moves sluggishly at the commencement of the inflammation; and, when effusion has taken place, its movements are entirely suspended, the preternatural connexions by adhesion concurring with the change of structure in producing this effect. The pupil, consequently, cannot exhibit the ordinary variations in size; it is contracted, and it becomes smaller and smaller in the progress of the affection. At the same time the effusions of lymph and the adhesions change the figure of the opening, rendering it angular, and often extremely irregular. Together with other changes, the pupil sometimes undergoes an alteration in situation, being apparently drawn upwards and inwards, or towards the root of the nose. It may deviate in other directions. The margin of the aperture is thickened, and has a villous or spongy appearance in the beginning of the disease, presenting a strong contrast to the thin, sharp, and well defined edge which naturally belongs to it. The effusion of lymph into the aperture, which has been already noticed, destroys its clear black colour, and gives it a dull cloudy appearance." 142.

*Increased Redness of the Eye.* Round the cornea is a red band, deeper coloured in front, and gradually shaded off behind, the circumference of the globe being comparatively clear. In the commencement of the affection, the sclerotica, as in all other cases in which it is inflamed, exhibits a pale pink redness, and the vascular trunks lying on the membrane are seen of a deeper pink tint under the unaltered conjunctiva. They advance in nearly straight lines from the circumference of the globe, ramifying towards the front, and are lost in the pink zone. The redness of the sclerotica and distention of its trunks increase as the affection proceeds. The vessels of the conjunctiva soon become partially enlarged towards the anterior part of the eye, and are distinguished by their scarlet colour; their fine close ramifications combine with the pink redness of the sclerotica to form the vascular zone round the cornea. The minute vessels terminate abruptly at the edge of the cornea, under which they probably pass to the iris. The zone varies in breadth; it is of a deep vivid red in acute iritis when fully developed; and in iritis of the most violent kind all the external vessels of the globe are equally affected, giving to it an uniform fiery redness. The red zone lasts as long as the inflammation of the iris, and is connected with it in its origin and decline. The whole iris is usually inflamed, but only one point may be so; and then the redness of the sclerotica is confined to the point opposite to it. In general inflammation of the iris, one part may be most acutely inflamed, and opposite that the external redness is greatest.

*State of the Cornea and Aqueous Humour.* From the vascular connexions between the sclerotica, cornea, and iris, a change might be anticipated in the state of the cornea from inflammation of the latter. General haziness occurs at first; nebulous opacity comes on when the inflammation is violent and long-continued. In most cases the cornea generally is affected; but partial opacity may exist with the general haziness or nebula. Rarely, there is ulceration of the cornea. These corneal affections add to the imperfection of sight caused by the changes in the pupil. No change in the state of the aqueous humour is discerned whilst the cornea remains clear: none can be discerned, when the cornea is hazy or opaque.

*Intolerance of Light and Pain.* There is generally some, and often considerable intolerance of light in the beginning, with lachrymation on exposure to it. These symptoms probably depend on the implication of the sclerotica, and they continue, although the quantity of light admitted into the eye is constantly diminishing in consequence of the changes produced in the pupil and cornea. There is generally more or less pain from the commencement, the degree varying according to the acuteness of the attack. It may be accompanied with burning sensation and tension, deep seated in the globe and orbit, extending to the head, and preventing rest entirely. On the other hand, the pain may be slight, even with considerable effusion of lymph and loss of sight. Patients often complain of great pain in the temple, brow, or cheek, as if it were seated in the bone. The pain is chiefly at night, and when present in the day it is exasperated at night time. Dimness of sight occurs in the commencement of iritis, and, as the changes in the pupil and cornea occur, vision becomes more imperfect or altogether destroyed.

*General Symptoms.* The constitutional disturbance is very various. Acute iritis may be attended with the symptoms of inflammatory fever, or the symptoms may be slight, nay entirely wanting.

"*Progress and Extension of the Inflammation.* If the inflammation, having attained its full development, should continue, the iris swells, or appears to swell; that is, it approaches towards the cornea, becoming convex in front, diminishing the anterior chamber, and sometimes having its surface puckered and irregular. Is this an actual swelling of the iris, real thickening of the part from interstitial deposition? or mere protrusion by the swelling of parts behind, by the effusion of lymph, or by aqueous secretion? Dissection has not yet elucidated these questions.

If the progress of the affection be not checked, it does not remain limited to its original seat in the iris. At first it appears on the very border of the pupil; then shews itself on the inner circle; and subsequently extends to the outer circle, presenting the combination of symptoms already described. Supposing it to go on without interruption, it passes from the ciliary circumference of the iris to the corpus ciliare, the choroid coat and retina, with increase of pain and fever, and ultimately with irrecoverable loss of vision, from change of structure in the retina. At the same time, the mischief is propagated forwards; the cornea becomes more opake, the conjunctiva more inflamed, and great external redness is added to all the other symptoms, so that the case, which was at first simple iritis, becomes ultimately ophthalmitis, or inflammation involving the external and internal tunics generally.

The question naturally occurs, whether the inflammation, when thus propagated to the posterior tunics, presents in them the same characters as in its original seat; that is, whether it is attended by effusion of lymph? I have never had an opportunity of dissecting an eye in this state of disease, nor are any such dissections recorded. The escape of lymph through the sclerotica, which has been already mentioned, and the bulging of the globe at some distance behind the cornea, in cases where it is disorganized by this inflammation, which certainly is not owing to suppuration, would lead us to suppose that the question ought to be answered in the affirmative. Sometimes the internal tunics suffer generally from the beginning; and vision is impaired, although the pupil may remain clear. The term iritis, implying that disease is confined to one texture, is not properly applied to such cases." 150.

*Acute and Chronic Iritis.* These are loose terms. Serious mischief may occur in a few days, or weeks may elapse without it. Extension of inflammation to the posterior tunics is most to be dreaded in acute iritis, but the chronic form is not exempt from this danger. The milder degree of inflammation may creep on to the ciliary body and adjacent parts, producing in them changes of structure capable of injuring or destroying sight.

*Effects of Iritis.* When the inflammation is at an end, under favourable circumstances, the iris may be completely restored, whether it has been simply discoloured, or effusion of lymph has taken place either in a thin stratum or tubercular masses.

*Adhesions of the Pupil.* The lymph thrown out in iritis speedily becomes organized, producing new formations of a permanent character.

"Thus, when the inner circle of the inflamed iris has regained its natural appearance by the progress of absorption, the edge of the pupil is found preternaturally fixed to the crystalline capsule. It may be closely attached at one or more points, the rest of the circle being free. More commonly the connexion is effected by slender threads, long enough to allow some motion; there may be many of these fringing the whole opening, or only one. Such adhesions are dark coloured; that is, they are of the same colour as the edge of the pupil or the uvea, partaking, like other adventitious formations, of the nature of the surface which produces them. Under suitable treatment, in an early stage, adhesions of the pupil are sometimes detached, leaving behind, at least in some instances, black marks on the capsule, which I believe are permanent. These marks escape notice in consequence of the blackness of the pupil; they are, however, sometimes detected on close examination with a strong light on the eye. I have seen a complete circular series of such marks, which I discovered while accidentally examining the eye with the sun shining upon it. The patient had laboured under iritis; and the pupil, which had been fixed to the capsule in its whole circumference, was completely liberated by the means employed. A tubercle of lymph effused on the edge of the pupil will produce a broader adhesion, fixing, perhaps, one third or one fourth of the circle. The changes now described must necessarily affect the figure and motions of the pupil: they often render it very irregular, and impair or destroy its motions. Mere alterations of figure are not injurious to vision; which is just as good with the most irregularly shaped pupil as with a circular one; and we often see perfect vision with great and permanent contraction of this aperture. It must be understood, of course, that the retina is uninjured, and that the pupil, however irregular or small, is clear." 155.

*Change of Texture, and Colour in the Iris.* If much general effusion has taken place and has proceeded uncontrolled for some weeks, the texture of the iris is permanently changed, being altered in colour, diminished in lustre, and confused in its fibrous texture. Sometimes it is marked with small dark specks; sometimes it is of a leaden hue. These changes concur with the adhesions of the pupil in lessening or destroying the motions of the part.

*Adventitious Membrane in the Pupil.* If the lymph thrown out on the crystalline capsule be not soon absorbed, it is organized and forms an opaque adventitious membrane adherent to the capsule and the pupil, and corresponding in dimension to the size of the pupil at the time of the effusion. The opacity is greatest in the centre. In the contracted pupil it fills the whole aperture, but when the edge of the iris is withdrawn, it is surrounded partially or entirely by a clear black margin, and the iris is attached to it by adhesions either close or in the form of short black threads. These adhesions sometimes divide the clear part of the pupil into small roundish or irregular apertures, when the pupil does not act under the influence of light, and the application of belladonna is usually necessary. If the effusion has only partially occupied the pupil and margin of the iris, the adventitious membrane will be found towards one side instead of the centre, the edge of the pupil being fixed to it, and thus drawn out of its regular line while the rest of the opening is natural. This state is attended with more or less injury to sight, but the patient may read a little by means of belladonna.

"*Closure of the Pupil.* When large effusion has taken place into the posterior chamber, it is organised into a dense opake substance, to which the entire circumference of the pupil is closely fixed, the opening itself being greatly contracted, or actually shut, and generally removed more or less from the centre of the iris. By this complete closure of the pupil, (*atresia iridis perfecta,*) the communication between the two chambers is destroyed, and the passage of light into the eye, almost entirely intercepted, with corresponding loss of sight. By means of the adventitious membrane thus produced, the uvea may be rendered generally adherent to the crystalline capsule ; and there may be a large anterior chamber : or the iris may have been previously pushed forwards and in contact with the cornea, so as to destroy the anterior chamber.

*Atrophy of the Globe, and Fluidity of the Vitreous Humour.* When large effusion has occurred into both chambers, and when lymph has been deposited behind the iris in such quantity as to cause bulging of the sclerotica, or to escape through that membrane and raise the conjunctiva into a swelling, it will be completely removed by absorption, when the inflammation has ceased. But the internal parts of the globe are so altered in structure, that it becomes flaccid, and reduced in size; (*atrophia bulbi.*) This change sometimes takes place after complete closure of the pupil. A fluid state of the vitreous humour (*synchisis*) and consequent softness of the globe may take place after acute syphilitic iritis of long standing, without shrinking in size or atrophy." 158.

*Impaired Vision.* When the inflammation has extended to the posterior tunics, it often leaves behind it impaired vision in various degrees, although it should have been arrested by proper treatment. This may take place in chronic cases as well as in acute. After an apparent cure, the eye remains preternaturally sensible on exposure to cold or damp, or after exertion. This is chiefly observed when the inflammation has been considerable, or has lasted long from neglect or injudicious treatment. Repeated and troublesome relapses may be the consequence.

*Diagnosis.* The tubercular depositions of lymph, the reddish-brown discolouration of the iris on its inner circle, the nocturnal exacerbations of the pain, the angular disfiguration of the pupil and its displacement towards the root of the nose, together with the previous existence of syphilis, and in most instances the concomitant existence of other syphilitic symptoms, clearly distinguish the venereal iritis. The local symptoms alone are not always sufficient, for we sometimes see merely a general discolouration of the part, such as might occur in idiopathic or arthritic iritis. The age of the patient and the previous and concomitant circumstances, tend to clear the difficulty. In idiopathic iritis there is either no distinct deposition, or it occurs as a bright yellow elevation out of the texture of the part, which increases to a certain size, then breaks and allows the escape of a yellow matter, which sinks to the bottom of the anterior chamber. Such abscesses are not seen in syphilitic iritis. In the arthritic species lymph is effused from the margin of the pupils, but not deposited in a distinct form, and the adhesions are generally white. Both in the idiopathic and arthritic iritis the pupil generally retains its circular figure and central position. Mr. Lawrence, however, has seen the effusion of reddish, brownish, or brownish-yellow lymph on the iris in several instances, both of children and infants, in whom no suspicion of syphilis could be entertained.

*Causes.* We must look on syphilitic iritis as a secondary symptom, and, until we can explain the occurrence of other secondary symptoms, we must remain in the same darkness respecting the nature of the connexion of this with the primary disease. It usually appears without any assignable cause, although cold, wet, and other external influences may excite it in those who are predisposed to it.

*Other Concomitant Syphilitic Diseases.* Sometimes it occurs alone, but usually it is accompanied by such other symptoms as eruptions, papular, scaly, tubercular, and pustular; ulceration of the throat and mouth; pains of the limbs; and swellings of the periosteum. As it belongs to the earlier class of secondary syphilitic affections, it sometimes shews itself, like the other symptoms of that class, before the primary disorder is cured.

*Syphilitic Iritis in Infants.* This is rarely seen; by Mr. Lawrence only twice. In one case there were excoriations and ulcerations round the anus; the iris was dull and dark-coloured; the pupil slightly contracted; and there was some redness of the sclerotica. In the other case, the father having had primary venereal sores before marriage, the child, in a few weeks after birth, had an eruption all over the body, wasted, and seemed on the point of dying. It got well under the use of mercury in very small quantities. In a few weeks more, severe inflammation of the eyes came on, mercury was employed, the inflammation was arrested, but the child remained blind. Both pupils were fixed and moderately contracted; an opaque body, not a cataract, was seen behind one; the other was clear; but both eyes were blind. Mr. Lawrence has seen syphilitic inflammation of the internal tunics occurring as a secondary symptom in conjunction with scaly eruption, after the infection of a chap on the hand by the contact of discharge from a sore in delivery.

*Is Iritis caused by the use of Mercury?* "An opinion has partially prevailed that the use of mercury is capable of producing iritis. Some have considered that syphilitic iritis, as well as other secondary symptoms, either are rendered more frequent and severe by the employment of this remedy, or owe their very existence to it; while others have spoken of iritis generally as being caused by it. I have seen no instance of iritis, of whatever kind, in which there has appeared to me any reason for ascribing the occurrence of the complaint to this cause. In nine of the cases related in this paper, iritis came on where no mercury had been taken previously to its appearance; and in some of them the complaint was severe, and produced consequences injurious to vision: in others mercury had been administered only in small quantity, and the mouth had not been made sore; and there is not one in the whole list in which the remedy had either been employed for a long time, or affected the system severely. Iritis occurred in some of the cases which had been treated by Mr. Rose and Dr. John Thomson without mercury. Dr. Ekström, of Stockholm, informed me that he had seen many similar instances in the patients of an institution where the use of mercury in syphilis had been entirely abandoned for a long time. Iritis took place in a woman who had contracted syphilis from suckling a diseased infant, and had taken no mercury." 166.

*Prognosis.* This is favourable when the affection is recent and confined to the iris; indeed we need not fear considerable changes when this only is affected, for very considerable effusion may be removed. We must examine carefully whether the posterior tunics are involved. The changes in the cornea and pupil may impair sight considerably, and yet it may be ultimately restored. Nay, even considerable impairment of vision is sometimes found where the cornea is clear, and the pupil not visibly obstructed, and yet the sense is recovered, so that even affection of the retina is not necessarily a ground of unfavourable prognosis. When the whole iris is changed in colour, with considerable contraction of the pupil and an opaque substance in it, with intense external redness, great and deep-seated pain, and complete extinction of sight; the case is hopeless. Mr. L. has not seen vision recovered, when there is large effusion behind the iris, particularly if it has occasioned bulging of the sclerotica, or made its way through it. Considerable imperfection of sight may be removed if the affection is recent, but cases differ so much that we can hardly speak of definite periods. We may fairly expect to arrest the inflammation and remove its effects, when the iritis has lasted a fortnight or three weeks. In one case active inflammation had gone on for six weeks, yet the recovery of sight was nearly perfect. We must take a combined view of the activity and duration of the inflammation before we decide on the probable termination. We often effect much good in cases that appeared desperate.

*Treatment.* We have three principal objects in view: to arrest the inflammation, prevent the further effusion of lymph and promote the absorption of that already poured out, and to prevent the dilatation of the pupil. These may be accomplished by antiphlogistic measures, the employment of mercury, and the use of belladonna.

Whenever the inflammation is acute, with constitutional disturbance, when we fear the extension of the inflammation to the posterior tunics, and more particularly if such has occurred, we must bleed generally and locally, purge, give salines with antimony and aperients, keep the body and the eye at rest, in short use the items of the antiphlogistic treatment. When the disorder is less violent, cupping or leeching will suffice; the latter may be advantageously employed in many instances which are not of the most acute kind. Whenever there is feverishness, particularly if the pulse is full and strong, general bleeding may be had recourse to, but the absence of such symptoms does not contra-indicate the practice. If the local complaint be serious and threaten mischief, the treatment may properly begin with venesection, if nothing forbids its employment. Neither should we hesitate to repeat the bleeding whenever the state of the part of the system calls for it.

Local applications are of comparatively little consequence; the patient's feelings must be our guide as to the use of warm or cold. Blisters are not applicable to the acute stage of the disease, especially if applied near the eye.

We now arrive at a very important subject for consideration, the exhibition of mercury. Antiphlogistic measures may have arrested the acute symptoms of the inflammation, but still the action of the capillary vessels too frequently continues, the effusion of lymph goes on, and destructive changes are in progress. We must give mercury, not as a purge, not in alterative doses, but so as to put the system under its influence. It then cuts short the inflammation, and stops the effusion of lymph, when that already thrown out will be absorbed. The redness of the eye diminishes, and sudden relief is felt; the lymph begins to lessen and is soon removed; the colour of the iris is restored last. The red zone round the cornea looks pale and soon disappears. Mercury to do this must be used freely. It should be commenced after the abstraction of blood locally or generally and after purging. The best form is, two, three, or four grains of calomel to one-fourth, one-third, or half a grain of opium every eight, six, or in urgent cases every four hours. The influence of the remedy will be soon perceived. Under particular circumstances blue pill, the hyd. c. cret., or mercurial friction may be employed instead of the calomel. In respect to the extent to which mercury should be carried, and the length of time that it should be continued, Mr. Lawrence observes that the more powerful its action on the system, the more effectual is its control over the disease. Sometimes a mild course may fail, when a more powerful influence will quickly do the business. Full salivation quickly produced cuts short recent disease, as by a charm. The remedy may then be suspended, and its effects allowed to subside slowly, which will take two or three weeks; it will not be necessary to give more mercury. In general, indeed, it is sufficient to make it sensible in the mouth. In cases of longer standing, we must persevere until the lymph is absorbed, until the natural colour of the iris returns, the red zone round the cornea is gone, and vision is restored. This will require four, six, in some instances eight weeks. A longer time is usually required in relapses and second than in first attacks.

It may naturally be asked, if iritis absolutely requires the exhibition of mercury? It certainly does not. It may run its course without any treatment, but then it produces effects more or less injurious to vision. Again, iritis may be controlled by common antiphlogistic means, but in general they are not to be depended on in arresting the effusion of lymph. Although the inflammation may not be violent, the pupil will contract, and lymph be thrown out, and although the disease may be checked and subside, leaving the organ apparently recovered, adhesions and adventitious membranes will permanently injure vision.

"During the time that I was surgeon to the London Ophthalmic Infirmary, I frequently saw patients who had been treated by common means, and in whom general disorganization of the iris, contracted, closed or partially adherent pupil, obstruction of that aperture by adventitious organizations, and loss or serious injury of sight, had resulted from inflammations that might have been checked by mercury, without leaving any permanent ill consequence. I may observe that iritis, of whatever kind, is an affection easily managed: that it rarely fails to yield to proper treatment, even when the case has been originally neglected; and that the serious effects just detailed are chargeable to injudicious management only. A strong contrast to such cases is afforded by those, in which mercury is properly administered; the cure in the latter being rapid and complete, and the occurrence of ptyalism being in general attended with the most decided improvement in all the symptoms. In this latter respect the action of mercury exerts a much more marked influence over the complaint than the loss of blood. These points are fully illustrated by Cases VII. to X., XII., XIV. to XVII., and XIX. to XXVIII. In these and in many other instances, which have come under my observation, the continued progress of the inflammation until the system was brought under the influence of mercury, the immediate cessation of the pain and the corresponding diminution of all the other symptoms as soon as the mercurial influence was established, have afforded the most unequivocal proof of the great power which the remedy possesses over the complaint." 178.

In viewing iritis and considering its treatment, the effusion of lymph and the importance of preventing or removing it form a prominent object of attention. Similar effusions and interstitial depositions in the serous membranes are productive of comparatively little evil. The effects of mercury are most marked, after active antiphlogistic treatment has failed. There are two parties; one supporting the antiphlogistic treatment alone: the other, mercury alone. Mr. Lawrence, as we have seen already, sides with neither. The result of his experience is, that the successive or combined employment of antiphlogistic means and mercury give the quickest relief and effect the most perfect cure. Mercury is certainly used with the greatest effect in the active period of the inflammation and acute form of the complaint. But it is as certainly given with advantage occasionally after the active period is gone by, when only the apparently permanent effects of inflammation remain, organized adhesions, and considerable imperfection of sight. In such cases it has essentially improved vision, and therefore it is best to give it a trial. As the circumstances are not urgent, the mercurial influence may be slowly produced, but the effect should be kept up for some weeks. As local applications, a solution of the oxymuriate of mercury and the red precipitate ointment have been used. They are inadmissible in the acute stage, and of no use, as mercurials, in the chronic. But when patients complain of severe pain over the orbit at night, six grains of the mercurial ointment, with two of finely powdered opium well rubbed in before the time at which the nocturnal pain is expected to recur, will generally prevent it. Mercurial frictions on the brow do not, however, arrest the inflammation as the internal use of the remedy does.

The third object which engages our attention is the artificial dilatation of the pupil, in order to preserve its natural figure and dimensions. Certain vegetables, by their action on the iris, produce this curious effect. The atropa belladonna, hyosciamus niger, lauro-cerasus, and datura stramonium, possess the power in question. The usual mode of proceeding is to rub the moistened extract on the brow, or drop a solution of it into the eye. The latter is the most efficacious; a scruple of the extract being rubbed down with an ounce of distilled water, the fluid filtered through linen, and two or three drops being introduced between the lids. Dr. Reisinger prefers a solution of the hyosciamine or atropia, the active narcotic principles obtained from the plants enumerated, to the other methods of dilating the pupil. He uses a solution of one grain of hyosciamine to a drachm of distilled water. In an old woman the pupil remained dilated for seven days after its application. When the organ is inflamed and painful, the moistened extract rubbed on the brow should be employed; under other circumstances the solution should be dropped into the eye. If we wish to produce the greatest influence in the quickest manner, we should employ both methods at the same time. The immediate effect of these narcotics is enlargement of the pupil, or, in other words, contraction of the iris, which loses its power of motion, and is said to be paralysed. The effect is usually produced in half an hour, and lasts for several hours, or even some days. It is not uniform in all individuals, being greater in proportion to the healthy state of the eye. A temporary amaurosis is produced by it, but no permanent injury to vision ensues. In this country belladonna is almost exclusively employed, and what is remarkable, its influence on the iris is not in the least degree diminished by use. Patients occasionally complain of undefined uneasiness after its application, but Mr. L. doubts how far this is justly attributable to it.

Adhesions of the pupil prevent its dilatation more or less completely according to their number and nature. A general and close attachment precludes it altogether, whilst partial adhesions only affect the part in which they are situated, and allow of dilatation elsewhere. When the adhesions are through the medium of long and slender threads, a limited degree of motion is allowed. Belladonna and other narcotics are capable of dilating the pupil in many instances where the iris is no longer affected by variations in the quantity of light. Its permanent condensation by effusion of lymph, under violent and uncontrolled inflammation, renders it altogether incapable of motion, and incapable of being acted on by narcotics. The artificial dilatation of the pupil must be combined with the use of mercury, in order to prevent the contraction to which there is such a tendency in iritis, for belladonna will not answer when the iris is highly inflamed. But the application, if used to the brow, does no harm, and may even be advantageous by preventing further contraction. If adhesions have already taken place, belladonna will, if the effusion be recent, elongate them, and sometimes separate them entirely, so as to liberate the pupillary margin. But to do this, it is necessary that the case be recent, and a full mercurial action be combined with the belladonna. Under these circumstances Mr. L. has seen the whole edge of the pupil detached from the capsule of the lens to which it had adhered, and the capsule has exhibited a circular arrangement of black spots, marking the number and situation of the adhesions. These marks are of dark colour, derived from the uvea, and, as far as Mr. Lawrence has seen, they are permanent.

If we cannot explain the exact modus operandi of mercury in iritis, it need not create astonishment. After all the experience of three centuries in syphilis the powers and effects of mercury are disputed. Certain it is, the mercurial influence is beneficial in the idiopathic, as well as in the syphilitic iritis, although its full influence is less decided. In the rheumatic form Mr. L. has employed it with decided advantage, and its more moderate employment in alterative doses is generally useful.

The transparency of the cornea renders the case of iritis particularly well adapted for studying the effects of mercury. We can observe the alterations effected by disease and by our treatment. We observe that mercury puts a stop to that increased action of the capillary vessels on which the effusion of lymph depends. It may readily be imagined that this fact must have attracted the notice of all who have watched in a philosophic manner the diseases of the eye; and it may be easily conceived, that the idea of employing mercury in other inflammations tending to the production of coagulated lymph would present itself to the minds of reflecting men. It has done so, and inflammation of the retina, whether acute or chronic; inflammation of the serous membranes, as the pericardium, pleura, and peritoneum; nay, according to Mr. Lawrence, phlegmonous inflammation of the thigh, yield more or less to the influence of mercury, when ordinary antiphlogistic treatment will fail. Whether it promotes absorption, except by arresting inflammation, appears to Mr. L. problematical. In fine, the powers of mercury in inflammation, and that of more tissues than the serous, are so great and so striking, that we cannot do more than glance at the subject at present. How much might be written on the use and abuse of the mineral now.

Mr. Carmichael, of Dublin, has lately recommended the employment of turpentine in iritis in cases where mercury is inadmissible. He gives it in ʒj. doses three times daily, its nauseous flavour being best disguised by almond emulsion. Two ounces of the conf. amygd. should be used to the half-pint of water, the residuum being removed by straining. The following is Mr. Carmichael's formula:—℞. Olei terebinth. rectif. ℥j., vitellum ovi unius. Iere simul. et adde gradatim, emulsionis amygdalarum, ʒiv., syrupi corticis aurantii, ℥ij., spiritus lavandulæ compositi, ʒiv., olei cinnamomi, gtt. tres vel quatuor. Sumat. coch. ij. max. ter die. In a few cases it has been necessary to increase the quantity of the turpentine to an ounce and half, or two ounces in the mixtures, the other ingredients being proportionally diminished, and the dose being a drachm and a half or two drachms. The strangury, so apt to follow, is obviated by infus. lini and mist. camph. but when very urgent, the turpentine may be suspended for a time. The tendency to acidity in the stomach, is relieved by the addition of ten or fifteen grains of the carbonate of soda to the eight ounces of the mixture. If inflammation runs high, local abstraction of blood may be necessary, as where mercury is used in general it is not required. The state of the bowels requires attention, and constipation must be prevented. Such are the opinions of Mr. Carmichael. Mr. Guthrie has made a trial of the remedy at the Eye Infirmary. In some cases it has succeeded admirably; in others it has been of little service; and in some unequal to the cure of the complaint. Mr. Lawrence has had no experience of it.

This concludes the history of syphilitic iritis, the most complete perhaps that has been offered to the British public. The manner of handling it is certainly monotonous, unnecessarily dry, and too tautological. But the matter is good, the information offered is derived from practical experience, and we must look on the whole as a contribution of no mean value to ophthalmic surgery. We are next presented with the details of twenty-nine cases. We have not space for many, but cannot refrain from noticing a few.

Case 1. (I.) *Acute syphilitic iritis with papular eruption—extensive and repeated effusions of lymph on the surface of the iris—atrophy of the globe.*

W. W. æt. 21, a stout healthy man, had a sore on the prepuce, in the middle of May, 1827, and soon afterwards a bubo in each groin. He took pills, the sore healed, the swellings subsided, but his mouth was not made sore. In six weeks eruptions appeared, and he consulted a quack doctor without benefit. About the 8th of August he began to have pain in the right eye, and on the 16th there were the usual symptoms of iritis. The sclerotica was of a bright pink hue round the cornea, while numerous turgid trunks were seen lying rather farther back—the conjunctival vessels were distended—the iris had a dull muddy appearance without any trace of the natural fibrous structure, the inner circle being reddish brown, whilst in the outer this tint was mixed with a dull yellowish colour—the pupillary margin was thickened and irregular—the cornea dull, and the anterior chamber generally cloudy—the eye painful, especially on exposure to light with epiphora—vision very imperfect. There were also pains in the shin-bones—a declining papular eruption over the body—and a few papulæ on the mucous membrane of the eye-lids, where they formed small yellow points as large as a pin's head. *Cal.* gr. ij., *opii* gr. $\frac{1}{4}$. 6*tis. hor. Moistened extract of belladonna to eye-brows—milk diet.*

On the 19th the patient was worse, the iris was darker-coloured, and the anterior chamber more obscure. *Hirud.* vj. *tempor.* On the 20th a mass of light-coloured lymph had been effused on the lower and outer part of the iris, which was more discoloured, and very irregular. The mouth was slightly affected by the mercury. On the 21st the mass of lymph had increased. *Mercurial liniment to be rubbed on the arms night and morning.* On the 23d the eruption was disappearing, and the pains in the bones were gone, but the eye was acutely painful, and the effused lymph was increased. The mouth was very sore. On the 27th another small effusion of lymph had taken place below the former, which was rather increased. On the 30th no abatement of the inflammation or pain ; one large brownish yellow mass of lymph occupied nearly the lower half of the iris. *Twelve leeches to the eye.* Great relief was experienced, and on the 4th Sept. the external redness of the eye was less, the pain was gone, and the mass of lymph was diminished. The pupil, dilated by belladonna, was quite misshapen by adhesions. On the 11th the lymph was almost entirely absorbed, the eye altogether much improved, the ptyalism severe. *Omr. infric. P. c. cal. et op. b. d. tantummodò—broth diet.* On the 15th he was allowed some meat and beer, and there was a relapse on the 18th. *Cal., et op.* 6*tis hor., Emp. canth. temp. dext. Continue meat and beer.* 21st. Effusion of lymph renewed in former situation. *Milk diet.* The blister produced no vesication, and next day the eye was worse. *Hirud.* xij.—*emp. canth. et postea cerat. sabinæ.* The blister and savine obviously aggravated the inflammation, the lymph was increased on the 25th, and there was a further deposition into the texture of the iris. Sixteen leeches were applied on the 26th, 27th, and 29th of Sept. with favourable results, the absorption of the lymph proceeded slowly, and on the 6th October, eighteen leeches were applied. On the 8th there was a relapse of inflammation attributed to cold. *Hirud.* xx. Next day the leeches were repeated, and inunction with the mercurial ointment commenced. The patient, however, grew dissatisfied, and resorted to an oculist. On the 7th March, 1828, he returned, having completely lost the sight of the right eye, which remained inflamed and painful. We need not mention the treatment in the interim. There was now considerable external redness, especially on the lower and outer part of the globe, where a collection of dark red vessels was seen. Nearly the whole anterior chamber was occupied by a quantity of lymph, which allowed only the upper part of the iris to be seen; it was of a reddish-brown appearance. There was a deep-seated aching in the globe, and the right orbit and temple were constantly painful. *Hirud.* xij., *tempor.*—*Ung. Hyd.*, ʒj., *nocte maneque infric.*—*Cal.* gr. ij., *Op.* gr. j., *bis die.* The mouth was made sore—the inflammation of the eye gradually subsided, and at the end of two months atrophy of the globe had commenced and was proceeding.

Case 2. (III.) *Syphilitic Iritis of both eyes—acute in the left, and terminating in atrophy.*

"Thomas Robinson, ætat. 19, of pallid complexion, and apparently strumous habit, thin and weak, had a sore as large as a sixpence on the outer surface of the prepuce, about six months ago. It healed under the application of the black wash, and the use of a few mercurial pills. He has had no other syphilitic symptoms. Ten weeks ago he found his eyes becoming very weak; they watered a good deal, and he was obliged to discontinue his work as a steel polisher. About the same time he received a blow on the left eye from a twisted piece of paper. He has completely lost the sight of the left eye for the last five weeks: vision of the right eye has been much impaired for the same time. The left eye has suffered serious change of structure from acute internal inflammation, which still continues, and has altered the figure of the globe by causing a bulging of the lower anterior part of the sclerotica. The iris and pupil are in close contact with the cornea; the pupil being contracted, but not filled by any opaque substance. At the lower part of the iris there is a considerable and irregular deposition of lymph, brownish, and apparently mixed with blood. On the prominent part of the sclerotica there is a round protrusion, equal to a small pea, of light brownish appearance, covered by conjunctiva. It seems to be a portion of lymph making its way through the sclerotic coat, and it leads to the supposition that the distention and bulging of the sclerotic coat, as well as the contact of the iris with the cornea, are caused by an internal deposition of lymph, similar to that which is effused upon the iris. The inflammation has extended to the outer tunics; there is considerable and general external redness, but the vascular trunks are largest and most numerous over the prominent portion of the sclerotica. The accompanying pain has not been in proportion to the vascular congestion, the effusion of lymph, and the disorganization: it has not interrupted rest, and is at present inconsiderable. The eye is absolutely insensible to light. No disorder is observed in the right eye on a superficial view; but closer examination detects in the iris and pupil effects of languid or indolent inflammation, the character and consequences of which are strongly contrasted with what has occurred in the other eye. The iris has in great measure lost its natural brilliancy and fibrous appearance, and its lower half has a slight yellow tint. The pupil, in point of size, is in about its middle state, and adherent throughout by a series of minute short dark filaments, which give it a fringed appearance. There is some redness round the margin of the cornea, and this is most conspicuous below, opposite the yellow discoloration of the iris. He can distinguish letters an eighth of an inch in length, with some difficulty, and cannot see any of a smaller size. He has had no pain, heat, nor uneasiness in this eye, nor any pain in the head or temples." 226.

He was cupped on the temples to 12 ozs. and had calomel and jalap.

On the 15th he was ordered *Cal.* gr. ij., *Opii*, gr. ⅓, *6tis hor.—Belladonna superciliis.* On the 21st, the mouth was decidedly affected. The left eye was much improved; the external redness was gone from the upper part of the globe, and the quantity of lymph was diminished. The appearance of the right eye was not much changed. *Cal. et Op.*, *bis die.* On the 25th the mouth was less affected, and there was little alteration in either eye. *Cal. et Op.*, *t. d.* On the 31st, the mouth was again sore. The left eye felt flaccid, and unresisting on pressure. On the 19th Jan. the left eye had shrunk considerably, the globe being so flaccid that it felt quite soft through the palpebræ; the lymph was completely removed from the anterior chamber, and the prominent tubercle on the sclerotica was absorbed. The right eye was quite natural, excepting the pupillary adhesions, but vision was still imperfect. The mercury was continued so as to affect the mouth, up to the 8th of February, when he was discharged. He could read the smallest print by day-light, but not so perfectly by candle-light. The left globe was considerably shrunk.

Case 3. (VII.) *Syphilitic Iritis of the left eye, without any other constitutional symptom.*

James Taylor, æt. 25, a stout healthy man, a paviour, had been salivated six or seven times for the venereal disease. In November, 1829, he had a sore on the penis, with buboes and eruptions. The mouth was affected for a short time with mercurial frictions, and he soon got well. Whilst in the hospital, with the mouth still sore, he was attacked with an inflammation of the eyes, which subsided under two cuppings and aperient medicines. He continued well till the 28th April, 1828, when he experienced dimness of sight in the left eye, soon followed by pain and inflammation. It became gradually worse, nothing was done for it, and he re-entered St. Bartholomew's Hospital, on the 5th of May. There was now considerable external redness—the iris was dull and discoloured throughout, and did not move on exposure to light, which caused no increase of pain nor lachrymal discharge—the pupil was partially dilated—the cornea was dull, with a general cloudy appearance in the anterior chamber—the patient suffered little in the day, but the pain in the eye prevented rest at night. He could not distinguish even large print with the left eye, and saw indistinctly with the right, which was affected sympathetically. *C. c. temp. sinist. ad* ℥xiv. *Cal. et Jal. Postea haust. purg. Ung. Hyd. fort.* gr. vi. *Pulv. opii*, gr. ij. *supercilio nocte infric.* On the 6th, he had 12 leeches, and was ordered *Cal. et Op.*, *6tis hor.* On the 8th he was cupped on the back of the neck to 16 ozs. On the 9th salivation had occurred and the eye was much improved. On the 1st of June he was discharged perfectly well.

Case 4. (XII.) *Syphilitic Iritis of both eyes, with primary sore and bubo, and papular eruption—no previous use of mercury.*

John Rose, æt. 20, of strumous diathesis, applied at St. Bartholomew's on the 27th October, 1825. There was a superficial healing ulcer on the prepuce, with a firm and rather indurated cicatrix, shewing the sore to have been originally more extensive—a healing ulcer, the size of a half-crown, in the groin—an eruption over the whole body of thinly scattered red pimples which suppurated slightly, and then declined, being most numerous on the face—well-marked iritis of both eyes, most advanced in the left. The irides, especially the left, were of a dull yellowish green; the pupils extremely irregular from slender adhesions; a considerable zone of external redness round the cornea, which in the left eye was hazy—vision greatly impaired, especially in the left eye—little pain in the eyes, and that only at night—skin and conjunctivæ slightly yellow—no fever. Three months previously he had contracted the sore behind the glans, and a bubo, which suppurated. Five weeks previously he had been confined by jaundice. The eruption and inflammation of the eyes had existed seven days. *Cal.* gr. ij., *Op.* gr. $\frac{1}{3}$, 6*tis hor. Ext. Belladon. palpeb. D. lact.*

On the 30th the mercury had produced no effect; the iritis was increased. The left cornea was very dull, and he could see nothing with that eye—severe pain in the head preventing sleep. *C. c. temp. sinist. ad* ℥xiv. *vel* xvi. *Cal. et Op.*, 4*tis horis.* The mercury affected the system in twenty-four hours, and the symptoms regularly and rapidly decreased. On the 3d Nov. the mouth was very sore. *To discontinue the mercury.* On the 6th, the primary sore was healed, and the induration removed; the eruption had disappeared, leaving only a few slight discolorations of the skin. Vision was restored. The irides had recovered their natural blueish-grey tint, the pupils were largely dilated and most of the adhesions had given way, the corneæ were perfectly clear, the red zone nearly gone. On the 9th the adhesions had nearly disappeared. On the 14th there was a slight blush of redness on the sclerotica of the left eye. *Pil. Hyd.*, gr. v. *t. d.* On the 16th the redness was increased, with slight pain in the eye. *Cal. et Op.*, 6*tis hor.* On the 18th the mouth was sore and the redness diminished. On the 21st the right pupil had again become adherent at two points. On the 25th the mercury was discontinued. Vision and the natural colour of the irides were restored; the pupil adhered at two or three points in each eye. On the 30th he was discharged cured.

Case 5. (VI.) *Syphilitic Iritis treated without mercury, and ending in contraction and adhesion of the pupil, with dimness of sight.*

"Mr. I., aged 22, of light hair and complexion, and robust habit, had an ulcer of the glans of moderate size, for which he took blue pill, so as to affect the mouth slightly. The sore healed in a fortnight, and he used the remedy about three weeks. There was no affection of the glands. Two months afterwards he had severe inflammation of the eyes, which first appeared in the right, and in a few days attacked the left also. It lasted about a month, and was attended with external redness, increased sensibility to light, lachrymation, and considerable pain; the latter coming on severely after he went to bed, lasting through the night so as to prevent sleep, and becoming less in the morning. No suspicion was entertained that this affection was syphilitic; no mercury was used, but active antiphlogistic measures were resorted to, including venesection, loss of blood by cupping three times, and numerous applications of leeches. When the inflammation had subsided, dimness of vision remained in the left eye, but the right recovered completely, although the pain had been most considerable in the latter.

In the course of a few weeks after the cessation of the inflammation, this gentleman consulted me on account of his left eye, in which I found a slight alteration in the colour and appearance of the iris, and several thread-like adhesions of the pupil, fixing the aperture in a state of contraction. He could see objects and even read in a good light, but found a mistiness or dimness before the eye.

He had also small copper-coloured scaly eruptions on the palms and fore-arms, and ulceration of the tonsils, although these symptoms had troubled him so little that he did not mention them to me, and I found them out only in consequence of questioning him on the subject. I ordered him two grains of calomel with one-third of a grain of opium thrice daily, and the solution of the extract of belladonna to be dropped into the eye. I saw him again at the end of a month, when I found that the mouth had been soon affected by the mercury, and that it still continued moderately sore. The eruptions and sore throat had entirely disappeared, and the dimness of vision in the left eye was nearly gone." 236.

CASE 6. (XI.) *Syphilitic Iritis of both eyes—mercury not previously used—one eye cured by the antiphlogistic treatment—mercury necessary for the other.*

John Durrant, æt. 22, a groom, admitted into St. Bartholomew's on the 23d June, 1829. Sclerotic of the right eye of a light pink hue on the front of the globe, the colour being deeper towards the margin of the cornea—iris dull and sluggish, the greater circle being nearly of natural colour, the lesser reddish brown or rusty coloured from the general effusion of lymph into its texture—edge of the pupil slightly thickened and villous, and adhering by a single brown thread—colour of the pupil natural—considerable pain in the eye, increased at night and on exposure to light—frequent epiphora—vessels of the conjunctiva partially turgid—cornea unaffected. He cannot even read a large print. The left eye exhibits similar appearances, but less in degree, and a single thread of adhesion appears in the pupil. A month or six weeks before Christmas he had gonorrhœa. About Christmas he had a small sore on the prepuce, and a swelling in the groin. He merely took some salts, and the symptoms disappeared in a month. At Easter he got wet, and rheumatism in the limbs, and cough and pain in the chest supervened, for which he was bled, and of which he gradually recovered. The affection of the eyes began three weeks ago, that of the right the first. Has had six leeches and a lotion, but no mercury. *C. c. tempor. ad* ℥vj.—*Cal. et Jal. Postea Pulv. purgans.*

On the 24th there was head-ache and feverishness. *V. S. ad* ℥xx.—*H. sal. antimoniat. 6tis hor.* 26th. *C. c. nuchæ ad* ℥xvj.—*Ext. Belladon. superciliis.* JULY 1. Right eye now well, and vision nearly perfect. The disease has advanced in the left eye, which is more painful. The belladonna always increases the pain, and must be left off. *Hirud.* xij. *oculo sinist.* On the 3d the inflammation was not diminished, although the strength was much reduced. A full papular eruption had appeared. *Cal.* gr. ij., *Op.* gr. $\frac{1}{3}$, *6tis hor.* The mercury at first caused purging, without affecting the mouth, but in the course of a fortnight ptyalism had been induced, and it was then discontinued. The natural appearance and powers of both eyes were completely restored, the eruption was nearly gone, and the health was much improved on the 22d. He was dismissed cured on the 1st of August.

CASE 7. (XV.) *Syphilitic Iritis, occurring during the use of mercury, and affecting both eyes in succession—very acute in the left eye—repeated relapses.*

Sarah Briant, æt. 17, a common street-walker, admitted into St. Bartholomew's Hospital, with vaginal discharge, condylomatous excrescences and sores about the anus, and an eruption half itch, half of scaly syphilitic character on the trunk and extremities. The sores had appeared about three months previously. The mouth had been made slightly sore by mercury. Common means were used with good effect, but on the 10th December the livid discolouration continuing, she was ordered *Pil. Hyd.* gr. v. *bis die.* On the 16th, the right eye was affected with iritis; there was increased vascularity of the sclerotica, general discolouration and dulness of the iris, and fixture of the pupil in about the middle state. On the preceding night she had suffered severe pain in the globe. *V. S. ad* ℥xvj. *Hirud.* xij. *P. c. Pil. Hyd. Fot. papav.* She was relieved by these means. On the 22d, all traces of the iritis were gone; the mercury was discontinued, the mouth having been sore for two or three days. A glandular swelling in the neck suppurated, and the patient was not discharged till Jan. 20th, the gums being still a little tender.

On the 30th, she was admitted with most acute inflammation of the left eye. It had commenced on the second day after her discharge, and rapidly increased—there was unremitting and most severe pain over the brow, and intense general head-ache. The sclerotica was almost of a violet tint—the cornea generally hazy—the anterior chamber cloudy—the iris dark and dull from general effusion into its texture, whilst a distinct mass of reddish lymph was deposited at its lower part near the pupil, which was motionless, irregular, and moderately contracted. *V. S. ad* ℥xxx. *Hirud.* xij. *Vesp. Cal. et Jal., postea haust. sennæ. Cal. et Op. 4tis hor.* Next day she was better. All the textures of the eye were less inflamed, and the lymph on the iris was a little diminished. The bleeding had produced faintness, and the blood had a firm buffy coat. The mercurial fetor was perceptible in the breath. *Hirud.* xij. *haust. sennæ.* On the 4th of February, a very marked improvement in the state of the eye had taken place. The inflammation of the conjunctiva and sclerotica was considerably diminished—the iris had nearly regained its natural colour, though a small quantity of lymph seemed still to remain where the mass was before observed, producing unequal dilatation under the use of the belladonna. Two days previously ptyalism had come on, and the calomel had been discontinued. *Moistened extract of belladonna to be smeared over the brow.* On the 6th, vision was much better. The pupil was more dilated, and exhibited three strongly marked irregularities from points of adhesion. On the 10th, she could distinguish small print with perfect ease, and the adhesions appeared to be giving way. On the 15th, vision was perfectly restored; the pupil was freely dilated by belladonna; the adhesions scarcely perceptible.

On the 19th, the eye was again inflamed. There was increased redness of the external tunics, and a considerable brownish discolouration of the iris at its inner circle. Vision was rather confused; no material pain in the globe. *C. c. tempor. ad* ℥xij. *haust. sennæ.* The eye was relieved, but hemicrania was complained of, and menstruation was deficient. *Pil. Al. c. Myrrh.* On the 27th, there was again a slight relapse of inflammation of the left eye, with considerable head-ache. *C. c. tempor. ad* ℥x. No blood was obtained, and on the 28th there was more discolouration of the iris. *Hirud.* xxiv. March 1. Head-ache more severe. *V. S. ad* ℥xiv. She was much relieved by this. On the 1st of April violent inflammation attacked the right eye, chiefly the sclerotica and iris, with great pain in the globe and in the head, and inflammatory fever. The left eye was slightly affected. *V.S. ad syncopen*, (42 oz.) The blood was buffed, and next day the symptoms were diminished. On the 3d April the improvement was still more decided. *Ht. sal. &c.* On the 6th, a relapse of inflammation. *V. S. ad* ℥xij. On the 8th, *hirud.* xij. *Emp. Canth. nuchæ.* The symptoms were very much relieved, but on the 15th there was a partial relapse in the same eye. On the 16th the inflammation was more severe. *Pil. Hyd. Sub. c. nocte maneque. Hirud.* xviij. *temp. dext.* She gradually recovered, and was discharged early in May. She was much reduced. Since leaving the hospital she has had three smart attacks of pain in the globe, but each yielded to a few leeches, a brisk purgative, and the subsequent use of quinine. The catamenia have in some measure returned, and she follows a laborious occupation without difficulty.

Case 8. (XXVII.) *Syphilitic Iritis treated ineffectually on the antiphlogistic plan, and immediately arrested by the use of mercury.*

"Ann Holly, aged 21, was admitted into St. Bartholomew's Hospital on the 9th of October, 1828, with gonorrhœa, large ulceration at the lower part of the entrance of the vagina, and a small indurated sore on the right nympha. On the 19th, she complained of pain in the left eye, which was a little redder than usual; six leeches were applied. The inflammation was more considerable the next day, and seated in the sclerotic coat: although there was no decided affection of the iris, I pointed it out to the pupils as being probably the very commencement of syphilitic iritis, and affording a favourable opportunity for trying the antiphlogistic plan. A large cupping was ordered from the temple, with an active purge of calomel and jalap. These means afforded no relief; and on the 22d, the iris had become dull, and sight was a little dim.—(Twelve leeches round the eye; two grains of calomel with one-third of a grain of opium every six hours.) No relief was experienced from the leeches; but the mercury acted on the system in forty-eight hours, and the affection was immediately checked. All appearance of inflammation had gone in four or five days, and she was discharged well on the 5th of November." 302.

Case 9. (XXV.) *Syphilitic Iritis—adhesions of the pupil, destroyed by the use of mercury and belladonna.*

Lucy Adams, æt. 20, admitted on the 21st Nov. 1828, with a small sore on each labium, and inflammation of the external tunics of the right eye; the sclerotica only was affected. The eye had been sore for three days. *C. c. temp. dext. ad* ℥xij. *Cal. et Jal. posteà haust. sennæ. Lot. Saturni. D. lact.* On the 24th, *hirud.* xij. *oculo. Pil. Hyd.* gr. v. *nocte maneque.* The inflammation of the eye gradually grew worse, and ended in a well-marked attack of syphilitic iritis. The iris was dull, discoloured, the pupillary margin was adherent in such a manner as to render the pupil transversely oblong. There was a pink zone round the cornea, pain in the eye, the brow and head, and dimness of vision. *C. c. temp. ad* ℥xij. *Cal.* gr. ij. *Op.* gr. $\frac{1}{3}$, 8vis *hor. Belladonna supercil.* The mercury acted quickly and powerfully, immediately arresting the adhesive inflammation of the iris. On the 1st December the effused lymph was completely absorbed, the iris had regained its natural colour and brilliancy, and the round figure of the pupil was restored. She was discharged on the 10th.

Case 10. (XXIX.) *Syphilitic Iritis, with other syphilitic affections, in an infant.*

"Jane Mudie, ætat. 26, and her female child, 16 months old, were admitted into St. Bartholomew's under my care, on the 31st of March, 1827. The mother had a discharge from the vagina with ardor urinæ three months before the birth of this child. The discharge continued till about three months ago, when she noticed sores on the external organs of generation. The labia, perineum, and verge of the anus are now occupied by elevated, warty, and ulcerated excrescences.

The infant, which was large and healthy at birth, had purulent inflammation of both eyes on the third day, and was taken to the London Ophthalmic Infirmary, where it soon recovered. At the age of five months a pustular eruption appeared on the neck, which soon went away. A discharge was then observed from the vagina; the labia swelled, and were excoriated; as this got better, flat warty excrescences appeared on the perineum and round the anus. Their surface is now ulcerated as in the mother.—(For the mother; pil. hydrarg. gr. v. night and morning: black wash. For the child; hydrarg. c. creta, gr. iv. twice daily; frequent ablution of the affected parts.) The symptoms, both of the mother and child, yielded to this treatment, and they were discharged well in about three weeks.

They were re-admitted on the 21st of May. The mother has now redness and swelling of the tongue at its apex, with an ulcerated fissure in the middle; and a superficial sore with yellowish surface, on the mucous membrane of the lower lip. These sores have existed for a fortnight: there is no return of disease about the external organs.—(Hydrarg. oxymur. gr. 3-8, in decoct. sarsap. comp. lb. i. daily.) The child has excoriations and ulcerations round the anus, which reappeared in a week after leaving the hospital. She has also, in the left eye, iritis in a mild form, which the mother ascribes to a cold caught by having the head wetted. It began three days ago. The iris has lost its brilliancy, and assumed a dark tint; the pupil is a little contracted; there is some redness of the sclerotica, and of the upper lid, and slight intolerance of light.—(Hydrarg. c. creta, gr. v. night and morning.)

24th. A small red and painful spot has appeared on the side of the tongue, near its basis, in the mother. The child's eye is worse; (three leeches.) The mild mercurial treatment above described was continued, both for the mother and child, till the 4th of June, when they were discharged, all symptoms having been completely removed. They were seen at the hospital some time afterwards, quite well." 307.

We now pass to the second division of this part of our able author's work. It treats of syphilitic ulceration of the eye-lids.

### II. SYPHILITIC ULCERATION OF THE EYE-LIDS.

1. *Syphilitic Eruptions of the Palpebræ.* Syphilitic eruptions, particularly the scaly and tubercular, frequently appear on the external surface, and on the ciliary margins of the lids. The latter are almost always red, excoriated, and sore in that form of syphilis imparted to an infant by a diseased mother or nurse, which is always confined to the skin. The mucous lining of the palpebræ sometimes participates in the syphilitic eruptions, but not so often as reasoning would lead us to expect. In Case 1, there were papulæ on the internal surface of the eye-lids. Some years ago our author had a gentleman under his care, with acute papular eruptions following chancre. The eruption extended to the mucous lining of the palpebræ, in which there were several pustules about as large as pins' heads, with some uneasiness and general swelling of the lids. They required no particular treatment—active antiphlogistic measures were employed. In August a scaly eruption appeared on the legs, the marks of the papulæ were still visible on the face; and the left upper lid was still red and rather swelled, the conjunctiva red and thickened, and the marks of the papulæ very evident. A patient was twice in Bartholomew's Hospital; first for phagedænic ulceration of the labia and one nympha, and subsequently for tubercular eruption, node of one tibia, and swelling of the upper eye-lid of one eye. The conjunctival lining was swollen, and an eruption of small pustules were observed upon it. She took calomel and opium freely, her symptoms quickly disappeared, and she was discharged cured.

2. *Syphilitic Ulceration of the Eye-lids.* It is not very rare, yet it is not described in works on syphilis. Our author's attention was first attracted to it many years ago by a case in Bartholomew's Hospital. A stout woman, who had long been on the town, was admitted with an ulcer which had nearly destroyed the lower eye-lid. The surface was greyish, with bloody points, the edge towards the cheek livid and sloughy, the discharge ichorous; the neighbouring parts were acutely inflamed and the face swollen; the sore and its vicinity acutely painful. There was no other venereal affection, local or general. Our author did not suspect syphilis, and employed leeches, fomentation, poultice, and opium without benefit, for the lid was completely destroyed. He now affected the mouth with calomel and opium, in two or three days the sore acquired a healthy surface, and cicatrization soon followed. He now entertains no doubt, that the ulcer was syphilitic. Soon afterwards Mr. L. had another case of a somewhat similar kind, and in the last few years he has seen so many, as to have learned that the progress and character of such sores are various in this as in other parts of the body.

The ulcer commencing on the ciliary margin, like a stye, may occupy the whole thickness of the lid, involving all its textures; or it may be confined to the external surface of the lid; or it may arise on the mucous surface and never extend beyond it. In one patient affected with syphilitic ulcers and periosteal swellings, the left upper eye-lid was red and swollen, and on its eversion there was seen on its inner surface a sore as large as a sixpence, with a tawny surface, and not reaching the edge of the lid. Mr. L. has seen several smaller sores at the same time on the mucous lining of both upper lids. The ulceration is sometimes acute, attended with inflammation and great pain, and rapidly destroying the affected part. In other cases there is little inflammation or pain, the disease proceeds slowly, and the cure is accomplished almost without loss of substance. The character of the sore will vary of course. The acute ulceration is of the phagedænic character, with red margin, sharp edge, foul unequal surface, on which bloody points are seen, and severe pain. In the chronic there is swelling and some hardness of the basis of the sore, with expansion of the cutaneous texture, instead of loss of substance, and little or no pain. Ulceration of the eye-lid generally occurs in conjunction with other syphilitic symptoms, such as ulcers in other parts of the body, and swelling of the bones or periosteum. In Case 1, the affection of the lid was the only secondary symptom for about two months, at the end of which time scaly eruption appeared. In other cases the eye-lid is the only part affected. A gentleman had a large ulcer, with dirty whitish surface, on the lining of the upper eye-lid. The character of the sore and the health of the patient made

Mr. Lawrence conclude that it was venereal, although he had not been affected with syphilis for a long time. The sore healed under the use of mercury and sarsaparilla.

The lower lid may be completely destroyed by ulceration, and yet the deformity may not be conspicuous; the upper lid, when the eye is shut, descending over and covering the globe. No other ulcerative affection of the palpebræ can well be confounded with that now described. In the great majority of instances the ulcers called cancerous begin in the integument and are for a long time confined to it, not reaching the ciliary margin or mucous surface until the disease has made some progress. It has two stages, the tubercular and the ulcerative. It begins with the formation of small, hard, and scarcely discoloured tubercles in the skin—these exist many years before ulceration takes place—it then proceeds slowly, the edge of the ulcer being hard and tuberculated, and several years elapsing without any great progress—the ulcer is gleety and has a scanty thin discharge, which forms an adherent scale on the surface. These cancerous ulcerations do not occur till the middle period of life and after it. The history of the case and its progress distinguishes the syphilitic ulceration.

*Treatment.* This may be summed up in a few words. Our author has tried mercury, found it answer, and been content. He has not experimented with sarsaparilla or other remedies. We pass to the cases detailed.

Case 1. (I.) *Indurated sore of the prepuce with phymosis—scaly eruption—large ulcer of the upper eye-lid.*

George Vaux, æt. 43, a copper-plate printer, a free liver, applied to Mr. Lawrence in August, 1829, with the left upper eye-lid thickened and elongated, and capable of being elevated partially only; its whole external surface covered by a circular sore, nearly an inch in diameter, encroaching on the ciliary margin, and, having destroyed a part of the eye-lashes; the sore considerably raised, its base and margin being thickened and rather indurated; no excavation of the surface, which was covered by a dry thin brownish scab. The sore and the surrounding skin were of reddish brown colour, the eye not inflamed, nor the sight injured. There was only a little smarting in the sore. Partial phymosis with copious yellow discharge—a hard lump felt on taking the prepuce between the finger and thumb, and an ulcer seen occupying the surface of the induration on forcibly retracting the prepuce. On the forehead were some coppery discolourations, and the same in other parts with a slightly scaly appearance. In March he had contracted gonorrhœa, and in a fortnight afterwards a sore appeared, which soon became obscured by the phymosis. In June the ulceration of the eye-lid commenced with a hard pimple on the margin of the lid amongst the lashes, which was lanced, soon got well, broke, and scabbed.

Under a poultice the scab came off, leaving a clean sore. On the 11th he was put upon calomel and opium, and on the 19th salivation had occurred. The hardness of the prepuce was diminished, the eruptions faded, the ulcer of the eye-lid healthy and cicatrizing. The mercury was taken less frequently. On the 26th the mercury was discontinued. The sore had nearly cicatrized, but had reached the mucous membrane; the eruption had disappeared; the sore on the prepuce had healed, and the discharge was gone. As the ptyalism subsided, the new cicatrix excoriated, and on the 9th September the ulcer was again spreading. Calomel and opium every night. On the 11th it was ordered twice daily, and on the 14th every eight hours. The mouth was again made sore, and pari passu, cicatrization of the sore advanced. The healing was accomplished by the gradual extension of new skin from the cutaneous margin of the ulcer to the mucous. On the 21st the mercury was ordered twice daily, and on the 23d it was discontinued, and on the 28th the patient was dismissed with a sore mouth. In the following May the thickening of the lid had disappeared entirely, the integument was natural, and the only mark left was a little deficiency in the ciliary margin and corresponding want of eye-lashes. Soon after this a tubercular eruption appeared on the scrotum, with superficial ulceration of the fauces, and imperfection of hearing. The lid continued well. The patient was put on blue pill.

CASE 2. (IV.) *Phagedenic Ulcer of the upper eye-lid without any other syphilitic symptoms—cured by mercury.*

"Louisa Williams, a fine healthy young woman, 25 years of age, was admitted into St. Bartholomew's Hospital, under the care of Mr. Earle, on the 14th of May, 1829. A phagedenic ulcer occupied the whole surface of the right upper eye-lid, and extended to both canthi; it had destroyed the external canthus, and the ciliary margin of the lid, and was extending along the conjunctival lining as well as externally. The surface of the ulcer was covered with a dry, brownish scab; the margin was inflamed, and acute pain was felt in the lid and over the brow. The patient asserted that she had never been affected with syphilis in any form, and referred the origin of the disease to a cold taken two weeks before, and followed by a stye, which broke, and gradually spread into a sore. When the scab had been removed by fomentation and poultice, lunar caustic was freely applied to the whole surface.

17th. The character of the sore is not changed; it continues very painful.—(Black wash, hydrarg. oxymur. gr. 1-8 in essent. sarsaparillæ, ℥ss. thrice daily.)

21st. The ulceration is spreading; the surface is ash-coloured, particularly at the margin, with an admixture of bloody points and streaks.—(Undiluted nitric acid to the parts last mentioned.)

I saw this patient on the 23d, with Mr. Earle, and found a large phagedenic ulcer of the lid, with inflamed margin, considerable and general inflammation of the conjunctiva, and great pain. I considered the disease decidedly syphilitic, and recommended that mercury should be employed so as to act speedily on the constitution.—(Two grains of calomel with one-third of a grain of opium every four hours.)

26th. The mouth is sore; the character of the ulcer is changed, and its progress is stopped.

31st. Cicatrization has proceeded very rapidly, and the patient leaves the hospital to-day, quite well." 332.

CASE 3. (V.) *Ulceration of the eye-lids, and other syphilitic affections in the eyes of two children.*

Sarah Coster, æt. 36, contracted the venereal disease from her husband about ten years ago. She discovered her complaint only a few days before the birth of her first child. She was cured in about seven months by mercury. Twelve months afterwards a sore broke out on the right thigh; she was again salivated, and got well in three or four months. A discharge from the vagina alone remained till seven or eight months ago, when the integuments over the head of the right tibia became inflamed and painful, and soon several small ulcers formed on the part. She was admitted for these under Mr. Earle, in July, 1827, and after a few weeks was discharged cured. In a fortnight the complaint broke out a-fresh, and gradually got worse till August 24, when she was re-admitted under our author. She had now phagedenic syphilitic ulcerations on the leg, and pain in the tibia, particularly at night. *Pil. Hyd.* gr. v. *nocte maneque—Lot. Cal. c. Hyd. Oxymur. Cat. panis.* On the 3d Sept. she left the hospital of her own accord, the leg being nearly healed.

*Children of Sarah Coster.* Her first child was a fine healthy boy at birth. A few days afterwards he had a severe attack of purulent ophthalmia in both eyes, which was cured in three weeks, and he has remained healthy.

Her second child, Sarah Coster, aged six, was also born healthy. A fortnight afterwards small healthy pimples broke out about the parts of generation, which were very red and sore. Patches of discolouration appeared about the face and head ; the skin generally was rough ; several of the nails separated leaving a scabby surface ; the nostrils were blocked up with a thick yellowish matter ; the eye-lids became inflamed and were agglutinated during sleep. She took grey powders (*hyd. c. cret. ?*) for several months, which caused soreness of the mouth, but did not cure her. She was in the hospital under Mr. Earle, in July, with sores on the lips, cheeks, and eye-lids; the latter alone remained sore when she went out with her mother.

The third child, Henry Coster, aged four, was born healthy and continued so till eight months ago, when the glands of the neck became enlarged and painful. He had also inflammation of the eyes, with great intolerance of light. He was cured in a few weeks and remained well for two months, when an eruption appeared over the whole body, with excoriation and foul ulceration about the anus and external parts of generation. These symptoms disappeared under the use of mild mercurial powders, when he was in the hospital with his mother in July. Soon after he left the hospital the eyes became inflamed, and a few spots appeared on different parts of the body. He re-entered the hospital with his mother on the 24th August. The right upper eye-lid was inflamed and swollen, and on everting it the mucous membrane was found to be occupied, in the whole extent of the tarsus, by a syphilitic ulcer with elevated edge and foul surface. The left upper eye-lid also was inflamed and slightly swollen, but its mucous surface was not ulcerated. There were a few discolourations and scaly eruptions on the head and trunk. *Hyd. c. Cret.* gr. v. *o. n. Ablut. tepid. tarsi.* The mother left the hospital in September, and the child accompanied her perfectly cured.

On the 4th October Sarah Coster was re-admitted, with her two children, Sarah and Henry. Her leg had got worse after she left the house, and now presented the same characters as before. Under small doses of the oxymuriate in the dec. sars. c. she was well enough to be discharged on the 26th November. The child, Sarah, had small superficial ulcerations about the mouth and on the mucous lining of the lips, and two rather larger sores on the cheek near the lobulus of the ear. The lids were red and slightly ulcerated on their margins. *Mild aperient medicine occasionally—ablution and simple applications.* The other child, Henry, had inflamed and slightly ulcerated eye-lids, with a small ulcer at the back of the neck. *Hyd. c. Creta,*

gr. v. *quotid.* In eight or ten days the mouth was affected, when the sores of the eye-lids healed. A large pustule formed both on the boy and girl, on the end of the right fore-finger, attended with considerable inflammation and pain. They left the hopital with the mother on the 26th November, and in March, 1828, the whole trio were reported sound,

This concludes Mr. Lawrence's work and our analysis. It is not our wont to indulge in long summings up. If we like a book we are careful to communicate its contents to the public; if we don't, we usually let it alone. This plan breeds fewest quarrels, and serves the interests of our readers very well. It is easy to indulge in sarcasm and invective, but it is not pleasant to experience or to witness their effects. By this simple rule the profession may ascertain our opinion of the present work. If we had not thought it good, we should not have taken such pains in our account of it. In short, it is a book which we conscientiously recommend to the notice of every man who would practice his profession with satisfaction to himself or advantage to his patient. Need we say more?

---

